# Endothelial Aquaporins and Hypomethylation: Potential Implications for Atherosclerosis and Cardiovascular Disease

**DOI:** 10.3390/ijms19010130

**Published:** 2018-01-03

**Authors:** Inês Vieira da Silva, Madalena Barroso, Teresa Moura, Rita Castro, Graça Soveral

**Affiliations:** 1 Research Institute for Medicines (iMed.ULisboa), Faculty of Pharmacy, Universidade de Lisboa, 1649-003 Lisboa, Portugal; imvsilva@ff.ul.pt (I.V.d.S.); madalenabarroso@ff.ulisboa.pt (M.B.); teresa@ff.ulisboa.pt (T.M.); 2 Department of Biochemistry and Human Biology, Faculty of Pharmacy, Universidade de Lisboa, 1649-003 Lisboa, Portugal; 3 University Children’s Hospital, University Medical Center Hamburg-Eppendorf, 20246 Hamburg, Germany; 4 Faculdade de Ciências e Tecnologia, Universidade Nova de Lisboa, 2829-516 Caparica, Portugal

**Keywords:** aquaporins, hypomethylation, S-adenosylhomocysteine, water and glycerol permeability, endothelial dysfunction

## Abstract

Aquaporins (AQPs) are transmembrane channels that facilitate water and glycerol permeation through cell membranes. Recently, the water channel AQP1 was suggested to contribute to endothelial homeostasis and cardiovascular health. Less is known about endothelial aquaglyceroporins expression and its implication in cardiovascular disease (CVD). We have previously used cultured human endothelial cells under a hypomethylating environment to study endothelial dysfunction and activation, a phenotype implicated in the establishment of atherosclerosis and CVD. Here, we used the same cell model to investigate aquaporin’s expression and function in healthy or pro-atherogenic phenotype. We first confirmed key features of endothelium dysfunction and activation in our cell model, including an augmented endothelial transmigration under hypomethylation. Subsequently, we found AQP1 and AQP3 to be the most predominant AQPs accounting for water and glycerol fluxes, respectively, in the healthy endothelium. Moreover, endothelial hypomethylation led to decreased levels of AQP1 and impaired water permeability without affecting AQP3 and glycerol permeability. Furthermore, TNF-α treatment-induced AQP1 downregulation suggesting that the inflammatory NF-κB signaling pathway mediates AQP1 transcriptional repression in a pro-atherogenic endothelium, a possibility that warrants further investigation. In conclusion, our results add further support to AQP1 as a candidate player in the setting of endothelial dysfunction and CVD.

## 1. Introduction

Aquaporins (AQPs) are a family of highly conserved transmembrane protein channels [1] that facilitate the transport of water and small non-charged molecules (such as glycerol) across cell membranes [2,3]. In humans, 13 isoforms (AQP0–12) were described with high amino acid sequence homology and tissue- and subcellular-specific localization [4,5] suggesting a link between the site of expression and physiological relevance [6].

AQPs are grouped based on their primary sequences and permeation selectivity. Orthodox AQPs are considered strict water channels (AQP0, 1, 2, 4, 5, 6, 8, although AQP6 and AQP8 are also involved in the transport of anions [7] ammonia [8]), whereas aquaglyceroporins facilitate the movement of glycerol and other small solutes in addition to water (AQP3, 7, 9, 10); unorthodox AQPs are found mostly intracellularly, with lower sequence homology and permeability not defined (AQP11, 12) [9,10].

Consistent with their wide tissue distribution, AQPs are associated with diverse physiological and pathophysiological processes [11]. Among the orthodox AQPs, AQP1 was found to be the major water channel expressed in endothelial membranes supporting a major role in transcellular water movement throughout the body [12,13]. Notably, the importance of AQP1 to maintain endothelial homeostasis and cardiovascular health has been recently reported [14,15]. Endothelial dysfunction is a well-established response to cardiovascular risk factors that precedes atherosclerosis development. It was documented that AQP1 is expressed in human atherosclerotic lesions and its deficiency was related to endothelial dysfunction and atherosclerosis progression [15]. In another example, illustrating the importance of AQPs in health, besides a strong involvement in gastrointestinal pathophysiology [16], aquaglyceroporins are emerging as key players in adipose tissue homeostasis and insulin response with potential implications in obesity and metabolic-related complications, such as metabolic syndrome and cardiovascular disease (CVD) [17]. Within the aquaglyceroporin subfamily, AQP7 and AQP9 are the most abundant glycerol channels in adipose tissue and liver respectively, and their coordinated regulation was shown to have a crucial role in maintaining body energy homeostasis [18,19]. During fasting, glycerol produced from triglyceride lipolysis in adipocytes is channeled by AQP7 into the bloodstream and is taken up by AQP9 in the liver for gluconeogenesis [20]. Knowing that the endothelium is a major regulator of tissue homeostasis and metabolite fluxes, endothelial glycerol channels may have a crucial role in tissue glycerol entry/exit through vascular endothelium. In fact, by immunohistochemistry analysis AQP7 expression was detected in capillaries of adipose tissue, cardiac and striated muscle [21,22] and an AQP10-variant expressed in the capillary endothelium in villi of the small intestine [23], suggesting an active involvement in glycerol absorption and distribution to tissues. AQP9 was also identified in pial vessels in the brain, where its main role was related to water transport through the blood-brain barrier [24]. However, besides capillary tissue expression, aquaglyceroporin subcellular transport properties in endothelia have not been investigated.

Increased concentrations of plasma homocysteine, or hyperhomocysteinemia negatively impact endothelial homeostasis and have been established as a common risk factor for CVD by mechanisms incompletely defined [25,26]. A current view holds that S-adenosylhomocysteine (SAH), the homocysteine precursor that accumulates in the setting of hyperhomocysteinemia, is the important mediator of homocysteine-associated atherosclerosis and CVD [27,28,29]. Supporting this possibility, our previous results clearly present SAH as a key player in the disruption of endothelial homeostasis that promotes cell hypomethylation and modulate epigenetic mechanisms, by compromising DNA and histone methylation [30,31,32,33]. We demonstrated a specific mechanism by which SAH-induced endothelial hypomethylation upregulates the expression of the adhesion molecules ICAM-1 (intercellular adhesion molecule-1) and VCAM-1 (vascular adhesion molecule-1) [32]. During atherogenesis, the inflammatory activation of endothelial cells is characterized by the upregulation of adhesion molecules, which by recruiting circulating leukocytes favor their trans-endothelial migration and atherosclerosis progression. In a series of studies, we demonstrated that SAH—induced upregulation of adhesion molecules promoted leukocyte adhesion [32]. Further, we documented the ability of excess SAH to activate endothelial prototypical pro-inflammatory signaling pathways and to upregulate the expression of several cytokines including TNF-α (tumor necrosis factor alpha) [33]. Moreover, our studies lead to the discovery that a specific methylation modification of histones is also decreased by endothelial hypomethylation contributing to endothelial dysfunction and activation. Interestingly, associations between altered methylation status of histone and changes in AQPs expression have been observed in different cell models [34,35].

In this study, we used human cultured endothelial cells to confirm the ability of excess SAH to trigger a pro-atherogenic phenotype and investigate the expression of several adhesion molecules at different cellular localizations, including at the cell surface. Subsequently, by performing a trans-endothelial cell migration assay, we attested the functional importance of SAH-mediated upregulation of adhesion molecules. Moreover, we measured cell membrane water and glycerol permeability in the presence and absence of SAH-induced hypomethylation. In order to ascertain a contribution of altered AQP-expression in impaired cell permeation and endothelial dysfunction, we evaluated the expression levels of several orthodox AQPs and aquaglyceroporins. Lastly, to gain insights into the potential molecular mechanisms that underlie the association between SAH-induced cell activation and altered AQPs expression, we investigated the role of exogenous TNF-α on the expression of the most abundant endothelial AQPs.

## 2. Results

### 2.1. SAH-Induced Endothelial Dysfunction

**Figure 1:**
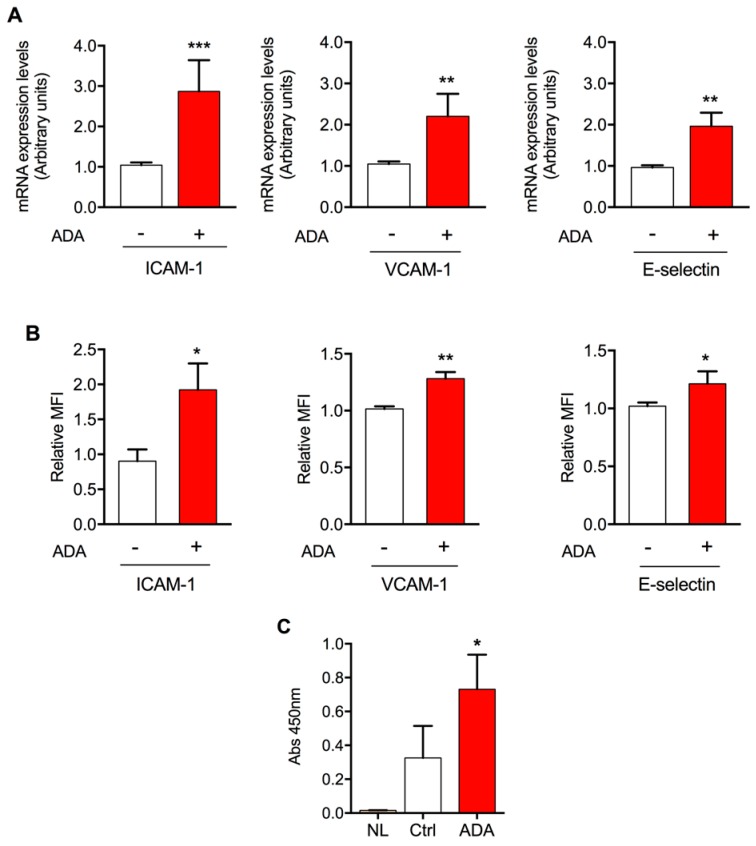
SAH accumulation and endothelial cell activation. (**A**)—mRNA expression levels of ICAM1, VCAM1 and SELE were measured by quantitative RT-PCR to determine the effects of ADA compared to control (*n* = 3–5). (**B**)—Flow cytometry was used to detect the relative cell-surface expression of ICAM-1, VCAM-1, and E-selectin, in ADA-treated and non-treated HUVEC (*n* = 3–6). MFI, mean fluorescence intensity. (**C**)—The effect of ADA treatment on the number of leukocytes that migrate through the endothelial monolayer was analyzed by a trans-endothelial migration colorimetric assay (*n* = 3). NL, no leukocytes added. Absorbance was measured at 450 nm. Data represent mean ± SD from three independent experiments. *, *p* < 0.05, **, *p* < 0.01, ***, *p* < 0.001, ADA versus non-treated cells.

Excess SAH induces cell hypomethylation. In order to promote SAH intracellular accumulation, the activity of the enzyme responsible for its production, SAH hydrolase, was inhibited with the adenosine analog, ADA. A strong intracellular accumulation (by more than 6-fold) of SAH in HUVEC exposed to ADA associated with a cell activation phenotype has been previously observed by us [30,32]. Here, and accordingly, ICAM-1, VCAM-1, and E-selectin mRNA levels were increased in ADA-treated cells (Figure 1A). Additionally, an upregulation of the same adhesion molecules at the cell surface was observed (2.1 ± 0.5, 1.3 ± 0.5, and 1.2 ± 0.1-fold increase of ICAM-1, VCAM-1, and E-selectin, respectively (*p* < 0.05) (Figure 1B).

Finally, to attest the functional importance of the SAH-mediated upregulation of adhesion molecules, we performed a trans-endothelial cell migration assay. Our results revealed a 2.2 ± 1.3-fold increase in the number of leukocytes that migrated through the endothelial cells when they were previously treated with ADA (*p* < 0.05 versus control; Figure 1C).

### 2.2. Effect of Hypomethylation on Water and Glycerol Permeability

A recent study showed that AQP1 is expressed in human atherosclerotic vascular lesions and its normal water channel permeability was considered important to promote cardiovascular protection by maintaining endothelial water homeostasis [15], but there are no reports regarding aquaglyceroporin and glycerol permeability. This prompted us to evaluate membrane permeability to water (P_f_) and to glycerol (P_gly_) in human endothelial cells, under normal conditions or ADA-induced hypomethylating environment. Permeability was assayed in individual adherent cells loaded with calcein under a fluorescence microscope as previously described [3] (see Methods).

**Figure 2:**
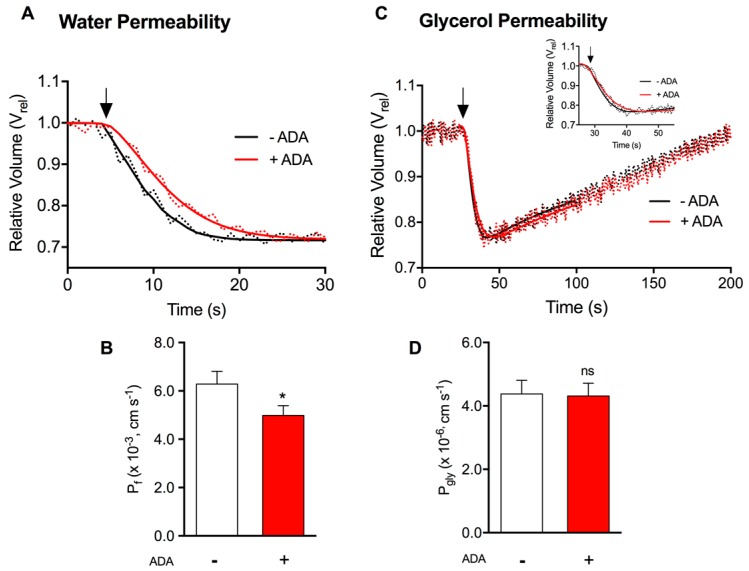
Water and glycerol transport in HUVEC under normal or hypomethylating environment. Water and glycerol permeability were assayed in adherent endothelial cells grown without (−) and with ADA (+). (**A**)—Time course of cell volume change caused by an osmotic challenge with mannitol (arrow) (*n* = 10). (**B**)—Water permeability (P_f_). (**C**)—Time course of cell volume change caused by an osmotic challenge with glycerol (arrow) (*n* = 10). Inset shows the first 50 s of cell volume change after glycerol addition. (**D**)—Glycerol permeability (P_gly_). Bars show mean ± SD from 10 cells analyzed on 3 coverslips in 2 cell platings. ns, not significant, *, *p* < 0.05 ADA versus non-treated cells.

When cells are exposed to a hyperosmotic mannitol buffer (impermeant solute), water outflow induces cell shrinkage (Figure 2A). Water permeability P_f_ is evaluated by monitoring the time course of fluorescence output that reflects the transient volume change (Vrel). As shown in Figure 2A, cells treated with ADA show slower rate of volume equilibration when compared with control cells. The calculated P_f_ values of cells in endothelial dysfunction condition ((4.99 ± 0.5) × 10^−3^ cm s^−1^) was significantly lower than control non-treated ((6.29 ± 0.6) × 10^−3^ cm s^−1^) (Figure 2B), suggesting that water channel proteins are involved in disease.

To assess glycerol permeability P_gly_, cells were challenged with a glycerol hyperosmotic solution and the time course of cell volume equilibration (*V*_rel_) was monitored. After the first fast cell shrinkage due to water outflow, glycerol influx is followed by water with consequent cell reswelling [3] (Figure 2C). ADA-treated and control cells showed similar rates of glycerol influx and no differences were detected. The calculated P_gly_ for cells in hypomethylation condition ((4.3 ± 0.5) × 10^−6^ cm s^−1^) was similar to that measured for control non-treated cells ((4.4 ± 0.5) × 10^−6^ cm s^−1^) (Figure 2D), indicating that SAH accumulation does not affect glycerol permeability. Though not affected by hypomethylation, these permeability values are within the range of the P_gly_ measured in other mammalian cell lines with high endogenous expression of aquaglyceroporin ((2 to 5) × 10^−6^ cm s^−1^), such as AQP7 and AQP3 in mouse adipocytes [36,37], rat pancreatic β-cells [38] and human epidermoid carcinoma cells [39] respectively, and contrasting with the low P_gly_ ((0 to 0.2) × 10^−6^ cm s^−1^) found in cells with residual aquaglyceroporin level [39,40]. Thus, the P_gly_ values evaluated may reflect the contribution of aquaporins in HUVEC for glycerol transport.

### 2.3. AQP3 Is the Main Endothelial Aquaglyceroporin

**Figure 3:**
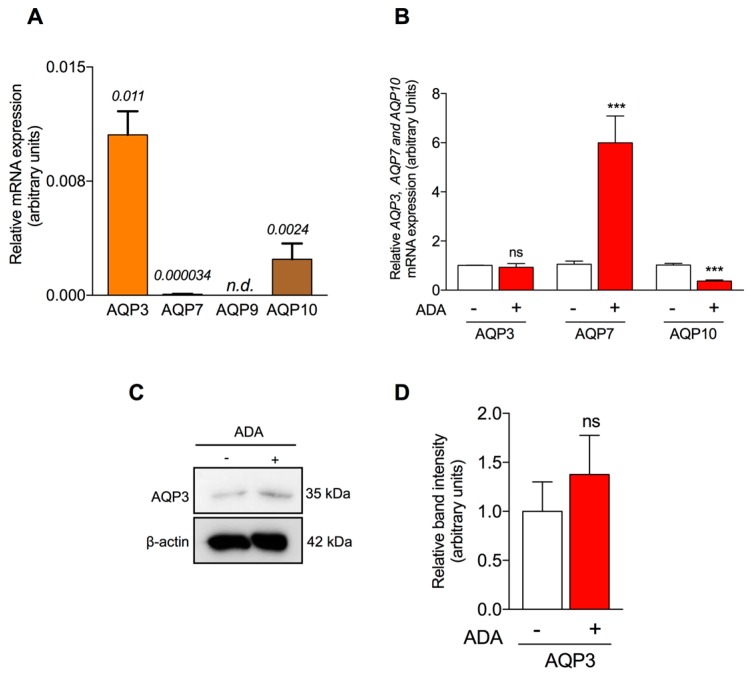
Effect of SAH accumulation on aquaglyceroporins expression in HUVEC. (**A**)—AQP3, AQP7, AQP9 and AQP10 mRNA expression levels. (**B**)—AQP3, AQP7 and AQP10 mRNA expression levels in endothelial cells grown without (−) or with ADA (+). Aquaporin mRNA levels are relative to β-actin mRNA levels and normalized to untreated expression for each AQP. (**C**)—Representative blots of AQP3 protein in endothelial cells obtained from HUVEC cultures non-treated (−) and treated (+) with ADA. (**D**)—Relative AQP3 protein expression. Data represent mean ± SD from three independent experiments. *n.d.*, not detected; ns, not significant, ***, *p* < 0.001 ADA versus non-treated cells.

To investigate if AQPs are involved in water and glycerol transport in human endothelial cells we first examined the gene expression levels of the aquaglyceroporins AQP3, 7, 9 and 10 in HUVEC (Figure 3A). Our results show that AQP3, AQP7 and AQP10 mRNAs, but not AQP9 mRNA, are present in HUVEC. Moreover, we found that AQP3 was the predominant aquaglyceroporin gene present in the human endothelium.

Under SAH accumulation, AQP3 mRNA expression was not affected (0.931 ± 0.1-fold), while AQP7, the less expressed aquaglyceroporin, was upregulated (6 ± 1.2-fold; *p* < 0.001) and AQP10 was downregulated (0.37 ± 0.02-fold; *p* < 0.001). Thus, the overall expression of aquaglyceroporins seems not to be affected by ADA, since AQP3 gene expression does not change and although AQP7 is upregulated, the opposing AQP10 dowregulation compensates the still very low AQP7 upregulated levels (Figure 3B). However, since we obtained P_gly_ values supporting aquaglyceroporin activity and AQP3 is the main aquaglyceroporin expressed in HUVEC, we subsequently evaluated AQP3 protein expression in control and ADA-treated cells (Figure 3C,D). The effect of ADA in AQP3 protein level (1.38 ± 0.6-fold) is in line with the AQP3 mRNA expression and discards any effect of SAH accumulation. Thus, our data show that AQP3 is the main glycerol channel in endothelial cells responsible for endothelium glycerol permeation; however, despite its physiological role in glycerol permeation, AQP3 may not be involved in the development of a vascular pathology.

### 2.4. AQP1 Is Implicated in Endothelial Dysfunction

**Figure 4:**
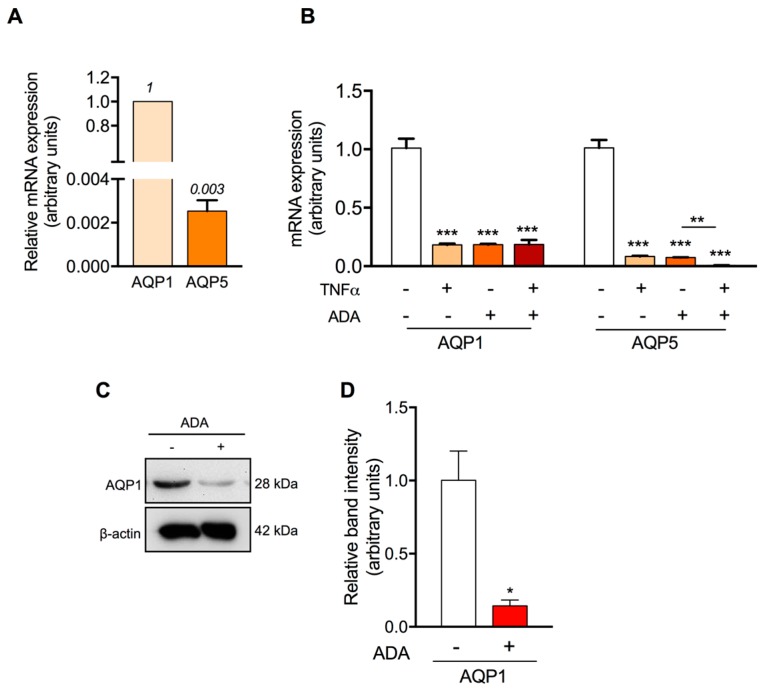
Effect of SAH and TNF-α in AQP1 and AQP5 expression. (**A**)—AQP1 and AQP5 mRNA expression levels in HUVEC. Aquaporin mRNA levels are relative to the mean of β-actin. (**B**)—AQP1 and AQP5 mRNA expression levels in endothelial cells grown without (−) or with ADA (+) and without (−), or with TNF-α (+). The effect of TNF-α was achieved by culturing the cells in the presence of 10 ng/mL TNF-α for 24 h. (**C**)—Representative blots showing AQP1 protein in endothelial cells obtained from HUVEC cultures non-treated (−) and treated (+) with ADA. (**D**)—Relative AQP1 protein expression. Data represent mean ± SD from three independent experiments. *, *p* < 0.05, **, *p* < 0.01, ***, *p* < 0.001 versus non-treated cells.

AQP1 was reported the most expressed aquaporin in human endothelial cells [13]. In view of the decreased P_f_ obtained when HUVEC cells were treated with ADA, we evaluated AQP1 mRNA and protein levels in ADA treated and control cells. In addition, we also investigated AQP5 mRNA expression in HUVEC since AQP5 is widely distributed in human tissues and its abnormal expression and function were correlated with several disease conditions [41]. Semi-quantitative gene expression of these AQPs (Figure 4A) confirmed AQP1 as the most expressed aquaporin in endothelium and also detected AQP5 mRNA expression although in a much lower amount. 

The pro-inflammatory mediator TNF-α is implicated in the regulation of the expression levels of the AQP1 and AQP5 [42,43]. Interestingly, we recently reported a link between ADA-induced hypomethylation and the increase of TNF-α levels in cultured human endothelial cells [33]. This led us to investigate the role of TNF-α in the ADA-induced downregulation of the AQP1 and AQP5 expression. The results, depicted in Figure 4B, show that TNF-α treatment downregulates the expression of both AQP1 and AQP5 (0.183 ± 0.01 and 0.083 ± 0.01-fold *p* < 0.001, respectively), as previously reported [42]. In addition, the expression levels of AQP1 and AQP5 were also drastically decreased under SAH accumulation conditions (0.184 ± 0.02 and 0.074 ± 0.01-fold; *p* < 0.001, respectively). However, co-incubation with TNF-α and ADA did not affect the already decreased AQP1 expression (0.187 ± 0.05-fold; *p* < 0.001), whereas AQP5 was further downregulated (0.0062 ± 0.002-fold; *p* < 0.001).

The role of AQP1 as main endothelial water channel was confirmed by evaluating AQP1 protein expression in HUVEC cells. As shown in Figure 4C,D, AQP1 protein expressed in control cells was dramatically reduced when cells were treated with ADA, in agreement with the obtained mRNA expression levels and with the measured water permeability in control and hypomethylation environment. These results strongly suggest that besides being the main water channel, AQP1 is involved in endothelial dysfunction.

## 3. Discussion

Aquaporins are crucial for the maintenance of tissue homeostasis. Previous studies have shown the involvement of AQPs in vascular pathophysiology. Recently, based on immunolabel-based detection approaches, a few aquaporin isoforms were found in mammalian vascular tissues [12,21,22,23,24] but their exact location in endothelial cells has not been ascertained. Here, by analyzing AQPs gene expression, we report for the first time that AQP3 is the most expressed aquaglyceroporin in primary human endothelial cells followed AQP10 and AQP7 in much lesser amounts. Accordingly, the permeability values for water and glycerol transport are in the range of those reported for other mammalian cells with high endogenous aquaglyceroporin expression [36,39].

This study was designed to investigate the potential involvement of aquaglyceroporins in endothelial dysfunction as a critical step in the development of vascular pathologies such as atherosclerosis. Impaired methylation has been associated with several diseases, including CVD [27,44,45,46,47]. Our previous studies using the same cell model have shown that an altered methylation status, due to excess SAH, promotes endothelial dysfunction by impairing nitric oxide (NO) bioavailability and by altering the cellular redox status [31,32,44]. Additionally, we found that excess SAH can lead to endothelial inflammation, inducing the expression of adhesion molecules and inflammatory cytokines [33]. Here, we examined the endothelial expression of key cell-surface proteins involved in the leukocyte adhesion and transmigration processes (Figure 1). ICAM-1, VCAM-1 and E-selectin contribute to the early stages of leukocyte adhesion [48]. We found that excess SAH increases the cell surface expression of these adhesion molecules, confirming our previous findings [32,33], that ICAM-1, VCAM-1 and E-selectin mRNA levels are increased in endothelial cells following ADA treatment. Moreover, we attested the functional importance of ADA-induced upregulation of adhesion molecules by performing a trans-endothelial migration assay that revealed a two-fold increase in the number of leucocytes that migrated through ADA treated cells, in comparison to non-treated cells. This observation discloses a new association between endothelial hypomethylation and augmented endothelial transmigration, a key feature of atherosclerosis development.

Water and glycerol permeability in ADA-treated and non-treated cells (Figure 2) was then studied. Our results show that endothelial hypomethylation is associated with altered water transport (20% decrease of P_f_), while glycerol permeability remains unaltered. This observation suggests that orthodox AQPs function and/or expression is impaired under endothelial hypomethylation, with potential implications for the establishment of a pro-atherogenic endothelial phenotype. As we found AQP1 and AQP3 to be the most predominant in HUVEC (Figure 3A and Figure 4A, respectively), we further assessed their expression under a hypomethylating environment. We observed a decreased expression of AQP1 but not of AQP3 in ADA-treated cells versus control (Figure 3B and Figure 4B, respectively). Importantly, there is a correlation between mRNA and protein levels for both AQPs (Figure 3 and Figure 4). In fact, AQP1 was previously reported to play a relevant role in the control of water permeability in endothelial cells [49]. Moreover, AQP3, although being a functional aquaglyceroporin that contributes to endothelium glycerol permeation, seems not to contribute to the development of ADA-induced endothelial dysfunction.

The observed changes in AQP1 expression in response to increased SAH levels may rely on altered transcription rates or mRNA stability. A recent study reported that AQP1 is intimately associated with non-activated, atheroprotected, endothelium whereas expression is absent in activated, ICAM-1 positive endothelium [14]. AQP1 expression is highly regulated and its promoter includes different regulatory elements, such as the atheroprotective transcription factor KLF2 [14], Sp1, AP1, AP2, and E-box elements [50]. It was reported that AQP1 expression can be reduced in the presence of TNF-α or IL-1β cytokines in rat endothelial cells [43], and that TNF-α might regulate AQP1 and AQP5 expression in a NF-κB dependent manner in human epithelial cells [42]. Interestingly, a previous study focused on AQP5 expression reported the first link between aquaporin expression and NF-κB activation [51]. The authors reported that TNF-α effect in AQP5 expression requires the nuclear translocation of NF-κB. The mechanism by which NF-κB activation downregulates AQP5 could not be clarified, however it was suggested that (i) a direct interaction with the AQP promoter might occur, where NF-κB may function as a transcriptional repressor. Alternatively, (ii) p50 and p65 NF-κB subunits can repress transcription by direct interaction with other transcription factors, such as SP1 [51]. 

Our group has previously reported that SAH-induced hypomethylation leads to NF-κB activation, which mediates the increased expression of adhesion molecules and inflammatory cytokines, as TNF-α and IL-1β [52]. In order to further dissect the mechanism behind SAH intracellular accumulation and AQP1 downregulation, we assessed the AQP1 and AQP5 endothelial expression in the presence of ADA, TNF-α, or both (Figure 4B). Independent treatments with ADA or TNF-α resulted in a similar effect, downregulation of both AQP1 and AQP5. However, co-incubation only showed a cumulative effect with AQP5, suggesting that ADA-mediated regulation of AQP1 may occur exclusively through TNF-α. Accordingly with our previous results, increased TNF-α in cells with increased SAH levels is dependent on NF-κB activation, but whether NF-κB mediates AQP1 transcriptional repression remains to be investigated.

Osmotic stress is one of the most challenging trials experienced by living cells and in order to maintain homeostasis, cells have to adapt fast when confronted with environmental perturbations. Aquaporins are among the mechanisms developed by living organisms to overcome these stresses. Since aquaporins are facilitators of membrane water permeation along with the much slower passive diffusion, and in endothelial dysfunction AQP1 is downregulated as a consequence of SAH accumulation and hypomethylation environment, we can infer that this disease condition renders cells more sensitive to osmotic stress. Notably, AQP1 was also reported to transport NO, the main anti-atherogenic molecule that plays a key role in the protection against the onset and progression of cardiovascular disease [53]. Therefore, AQP1 downregulation impact on endothelial function may go beyond osmotic transport impairment and further studies are required to better clarify its role in vascular disease.

## 4. Materials and Methods

### 4.1. Cell Culture and Treatments

Human umbilical vein endothelial cells (HUVECs) obtained from Lonza (Walkersville, MD, USA) were cultured at 37 °C in 5% CO_2_. Cells were grown in EGM-2 supplemented medium (Lonza). Experiments were performed between passages two and five and with cells 70% to 80% confluent. Treatments with adenosine-2′,3′-dialdehyde (ADA; Sigma-Aldrich, St. Louis, MO, USA) were performed for 48 h at 20 μM as previously optimized [54]. Tumor Necrosis Factor-α (TNF-α) treatments (10 ng/mL; Sigma) were performed for 24 h.

### 4.2. RNA Extraction and Quantitative PCR 

Total RNA was isolated using a RNeasy mini kit (Qiagen, Hilden, Germany) and treated with DNase I (Invitrogen, Thermo Fisher Scientific, Waltham, MA, USA). cDNA was obtained from 500 ng of total RNA and the retrotranscription reaction was carried out with SuperScript III kit (Invitrogen). Real-time PCR reactions were carried out using a CFX96 Real-Time System C1000 (BioRad, Hercules, CA, USA), the TaqMan Universal PCR Master Mix (Applied Biosystems, Thermo Fisher Scientific, Waltham, MA, USA) and the following specific TaqMan pre-designed gene expression primers: AQP1 (Hs01028916_m1), AQP3 (Hs01105469_g1), AQP5 (Hs00387048_m1), AQP7 (Hs00357359_m1), AQP9 (Hs00175573_m1), ACTB (Hs99999903_m1), ICAM-1 (Hs00164932_m1), VCAM-1 (Hs00365485_m1) and SELE (Hs00950401_m1) (Applied Biosystems). The ΔΔ*C*_t_ method was used for relative quantification of gene expression, using β-actin and glyceraldehyde-3-phosphate dehydrogenase (GAPDH) as endogenous controls.

### 4.3. Flow Cytometry

Flow cytometry was used to assess the expression of adhesion molecules in the cell surface. Adhesion molecules were stained independently with specific fluorescent-conjugated antibodies for ICAM-1 (SantaCruz Biotechnology, Dallas, TX, USA), VCAM-1 (R&D Systems, Minneapolis, MN, USA) and E-selectin (BioLegend, San Diego, CA, USA). Briefly, cells were trypsinized, washed with PBS, incubated for 30 min with specific antibodies, washed with PBS containing 0.1% NaN_3_, and analyzed using GUAVA EasyCyte 5HT FACS and InCyte software (Millipore, Burlington, MA, USA).

### 4.4. Transmigration Assay

Transendothelial migration of leukocytes was assessed using the QCM™ leukocyte transendothelial migration colorimetric assay (Millipore). Briefly, cells (80–90% confluent) were cultured in the presence or absence of ADA, for 24 h, and then replaced onto fibronectin-coated cell culture inserts (3 μm pore size) maintaining culture conditions. After 24 h, fresh leukocytes isolated from healthy individuals, that were processed with an RBC lysis solution (BioLegend) and re-suspended in DMEM media (Life Technologies, Thermo Fisher Scientific, Waltham, MA, USA) containing 0.5% BSA (Sigma), were added to the insert, on the top of the confluent HUVEC. After 8 h of incubation, the leukocytes in the lower chamber, which migrated across the endothelial cell monolayer, were stained accordingly to assay instructions and measured at 450 nm using an Asys Expert Plus plate reader (Biochrom, Cambridge, UK).

### 4.5. Western Blotting

Proteins were separated by SDS-PAGE before semi-dry transfer. After blocking, the following specific primary antibodies were used: anti-AQP1 and AQP3 antibody (1:100 and 1:200, respectively; Santa Cruz Biotechnology, Dallas, TX, USA), and anti-β-actin (1:1000; Sigma-Aldrich, St. Louis, MO, USA). Secondary anti-rabbit and anti-mouse (1:5000; Jackson Immuno Research, Newmarket, UK) and anti-goat (1:7500; Jackson Immuno Research) HRP-linked antibodies were used for ECL-mediated (Amersham Biosciences, Little Chalfont, UK) detection. Band intensity was measured using the ImageJ software (Available online: https://imagej.nih.gov).

### 4.6. Permeability Assays

Water (P_f_) and glycerol (P_gly_) permeability were measured in individual adherent cells on a coverslip, as previously described [37,55]. Briefly, cells were loaded with 5 mM calcein acetoxymethyl ester (calcein-AM; Sigma-Aldrich) for 30 min at 37 °C in 5% CO_2_. The coverslips with the adhered cells were mounted in a closed perfusion chamber (Warner Instruments, Hamden, CT, USA) on the stage of a Zeiss Axiovert 200 inverted microscope. Fluorescence was excited at wavelength 495/10 nm and the emission fluorescence collected with a 535/25 nm bandpass filter coupled with a 515 nm dichroic beam splitter. Images were captured using a 40× epifluorescence oil immersion objective and a digital camera (CoolSNAP EZ, Photometrics, Huntington Beach, CA, USA) and recorded by the Metafluor Software (Molecular Devices, Sunnyvale, CA, USA).

Cells were perfused with 300 mM HEPES buffer (135 mM NaCl, 5 mM KCl, 2.5 mM CaCl_2_, 1.2 mM MgCl_2_, 10 mM d-Glucose, 5 mM HEPES, pH 7.4, 300 mOsM) for 60 s, after which 300 mM mannitol (for water permeability) or 300 mM glycerol (for glycerol permeability) was added to the buffer to achieve an external osmolarity of 600 mOsM. Cell volume (*V*) was measured at selected time points from 2D images obtained during the permeability assays to evaluate the initial volume (prior to the osmotic challenge *V*_o_) and the final equilibrium volume [37]. P_f_ and P_gly_ coefficients were evaluated from the measured time dependent volume changes, *V*_rel_ = *V*/*V*_o_, obtained by adding mannitol (P_f_) or glycerol (P_gly_) to the external media, using the model equations described by Madeira et al. [36] and the Berkeley Madonna software (Available online: http://www.berkeleymadonna.com).

### 4.7. Statistical Analysis

Results were expressed as mean ± SD of *n* individual experiments. Statistical analysis between groups was performed by Student’s *t*-test. *p* Values < 0.05 were considered statistical significant. Statistical analyses were performed using the Graph Prism software (GraphPad Software, La Jolla, CA, USA).

## 5. Conclusions

Reduced AQP1 levels were previously associated with different CVD features, such as ischemia, hypoxia, and cardioplegia [52]. Our results support the involvement of impaired AQP1-mediated water transport in endothelial dysfunction and activation.

The homocysteine precursor, SAH, is once more shown to be an important player in endothelial homeostasis regulation. Our results suggest an additional mechanism through which SAH-induced hypomethylation environment may contribute to an endothelial pro-atherogenic phenotype, further supporting its causal role in vascular pathology.

We have found that increased SAH in human endothelial cells leads to decreased AQP1 levels and altered water permeability, with no effects on AQP3 levels and glycerol permeability. Furthermore, our results show that TNF-α treatment downregulates AQP1 expression in endothelial cells, suggesting that SAH-induced inflammatory activation might be responsible for the observed SAH-mediated decrease of AQP1. Although further investigation is needed to validate this hypothesis, our results suggest that AQP1 is a candidate player in the setting of endothelial dysfunction.

## Abbreviations

ADA	Adenosine-2′,3′-dialdehyde
AQP	Aquaporin
CVD	Cardiovascular disease
ICAM-1	Intercellular adhesion molecule-1
NF-κB	Nuclear factor-κB
NO	Nitric oxide
SAH	S-adenosylhomocysteine
TNF-α	Tumor necrosis factor α
VCAM-1	Vascular cell adhesion molecule-1
